# Clinical Pathologic Profiles of *Helicobacter pylori* Reveal Age-Specific Peaking with Concomitant Chronic Gastric Inflammation, Robust Immunity, and Tissue Alterations Implying Potential Predisposition to Malignancy in Ha’il, Saudi Arabia

**DOI:** 10.3390/jcm14082643

**Published:** 2025-04-11

**Authors:** Kamaleldin B. Said, Khalid F. Alshammari, Safia Moussa, Ruba M. Elsaid Ahmed, Ahmed H. Aljadani, Najd B. Albalawi, Layan Al-Hujaili, Ruaa Alharbi, Arwa A. Alotaibi, Fahad M. Alshammary, Fayez R. Alfouzan, Zaid A. Albayih, Bader I. Alkharisi, Ghadah N. Alsdairi, Shumukh H. Alshubrami

**Affiliations:** 1 Department of Pathology, College of Medicine, University of Ha’il, Ha’il 55462, Saudi Arabia; 2 Department of Internal Medicine, College of Medicine, University of Ha’il, Ha’il 55462, Saudi Arabia; 3 Department of Clinical Microbiology, King Salman Specialist Hospital Ha’il 55462, Saudi Arabia; sali36@moh.gov.sa (S.M.);; 4 Family Medicine Resident, Ha’il Health Cluster, Ha’il 55462, Saudi Arabia

**Keywords:** *Helicobacter pylori*, chronic gastritis, prevalence, gastrointestinal pathologies, histopathology

## Abstract

**Background/Objectives:** *Helicobacter pylori* (*H. pylori*) is a significant global health issue causing chronic gastritis, peptic ulcers, and gastric malignancies. Unfortunately, many, particularly in the Middle East, continue to exhibit alarming rates of prevalence. This study aimed to elucidate local epidemiological patterns of *H. pylori* and examine its histopathological impact on the gastric mucosa. **Methods:** This retrospective-cross-sectional study included 805 symptomatic adults (329 males, 476 females) who underwent endoscopic evaluation at King Salman Hospital, Ha’il, Saudi Arabia. Biopsies from the antrum and body were processed using routine formalin fixation and paraffin embedding. Staining with hematoxylin–eosin (H&E) and Giemsa permitted assessment of chronic gastritis and detection of *H. pylori*. Data were evaluated by IBM SPSS (version 23, IBM Corp., Armonk, NY) for associations among infection, histopathology, and patient characteristics. **Results:** A total of 727 (90.3%) were *H. pylori*-positive with marginally higher rates in females (91.2%) than males (89.0%). Infection spanned all age groups, reaching 100% in males aged 60–80 years. Overall chronic GI complications were identified in 726 (99.9%), with chronic gastritis being the most profound histopathologically (19.3%). Lymphoid aggregates in 93.0% biopsies reflected a pronounced immune response. Advanced lesions, including metaplasia (0.8%), atrophy (0.3%), and lymphoma (0.1%), were uncommon, though indicative of potential malignant progression. Despite both sexes exhibiting universal symptoms of gastritis, dyspepsia, and heartburn, there were no statistically significant gender-based differences (*p* > 0.05); specifically, post-*H. pylori* signs such as vomiting, nausea, weight loss, bleeding or hematemesis occurred equally in all. Histopathology consistently revealed chronic active gastritis with glandular distortion, lymphoplasmacytic infiltration, and occasional mucosal erosions. Giemsa staining further confirmed abundant spiral shapes underscoring a high bacterial load. **Conclusion:** These findings highlight the age-specific persistently elevating rates of *H. pylori* significantly associated with chronic gastric inflammatory complications. Although advanced gastric lesions remain rare, reflecting regional epidemiology, early screening, and sleeve treatment efforts, the potential for malignant transformation makes it imperative for continued vigorous eradication, therapy, and vigilant follow-up to avert severe disease outcomes.

## 1. Introduction

*Helicobacter pylori* (*H. pylori*) infection remains one of the most prevalent bacterial infections globally, affecting approximately 43.9% of the adult population as of 2015–2022, marking a decline from 52.6% before 1990 [1]. This Gram-negative, spiral-shaped bacterium has evolved to survive in the harsh acidic environment of the stomach, challenging the prior assumption that the stomach was a sterile organ. Its discovery in the early 1980s revolutionized the medical understanding of gastrointestinal diseases and their underlying causes [2].

*H. pylori* infection is chronic, often persisting lifelong if left untreated, and universally leads to chronic gastritis, characterized by persistent inflammation of the gastric mucosa. While frequently asymptomatic, chronic gastritis associated with *H. pylori* can escalate into severe pathological conditions, including peptic ulcers, gastric adenocarcinomas, and mucosa-associated lymphoid tissue (MALT) lymphomas. Globally, *H. pylori* is responsible for approximately 90–95% of duodenal ulcers and 70–85% of gastric ulcers, signifying its pivotal role in peptic ulcer disease [3,4].

The worldwide burden of *H. pylori*-associated diseases remains substantial, with nearly half of the adult population infected, equating to about 4.4 billion people. Epidemiological estimates indicate that approximately 48.6% of the global adult population harbors *H. pylori*, with prevalence being significantly higher in developing countries compared with industrialized nations [1]. Notably, the Middle East and North Africa (MENA) region demonstrates substantial variability in *H. pylori* infection rates, with prevalence among adults ranging widely from 36.8% to 94% across different countries and localities. In Saudi Arabia specifically, regional differences are particularly pronounced, with estimates ranging dramatically from 10.2% to 96%, reflecting substantial variations in socioeconomic factors, hygiene practices, and public health awareness [5,6,7].

Aside from its established connection with gastrointestinal pathology, *H. pylori* infection is associated with increased risks of extra gastroduodenal diseases, including hematological, autoimmune, metabolic, cardiovascular, neurological, and allergic disorders [8]. Notably, *H. pylori*-related chronic gastritis is recognized as a precursor lesion in Correa’s cascade, progressing through atrophic gastritis, intestinal metaplasia, dysplasia, and ultimately gastric adenocarcinoma—a malignancy responsible for significant global morbidity and mortality [9]. Indeed, the International Agency for Research on Cancer (IARC) classified *H. pylori* as a Class I carcinogen, highlighting its critical significance in gastric oncogenesis [10,11]. The mechanisms underlying these associations often involve systemic inflammation and immune dysregulation triggered by *H. pylori*. Autoimmune diseases, in particular, are increasingly linked to this bacterium due to mechanisms like molecular mimicry and the induction of autoantibody production. Notably, approximately 43% of autoimmune patients reportedly have concurrent *H. pylori* infection [12,13,14].

Given the extensive health burden associated with *H. pylori*, accurate and timely diagnosis have significantly enhanced diagnostic accuracy [15,16]. Treatment strategies typically involve combination therapies consisting of antibiotics and proton pump inhibitors to enhance efficacy. However, growing resistance to commonly used antibiotics has complicated treatment regimens, necessitating tailored therapeutic strategies based on antimicrobial susceptibility testing. Considering the high prevalence and significant clinical impact of *H. pylori* infection globally and particularly within the MENA region, there remains a substantial gap in public awareness and understanding of this bacterium and its associated health risks.

The current study aimed to investigate the epidemiology, clinical presentations, and histopathological features associated with *H. pylori* infection in a regional context, elucidating factors influencing the infection’s prevalence and severity. By thoroughly exploring the relationship between *H. pylori* infection, gastrointestinal and extragastric diseases, and assessing diagnostic efficacy, this research contributes valuable insights toward refining clinical management strategies, mitigating disease progression, and ultimately reducing associated morbidity and mortality. The findings from this research could provide crucial guidance for public health policy-makers and healthcare practitioners, particularly in regions with high infection prevalence, enabling more effective interventions and resource allocation to combat *H. pylori*-related diseases globally.

## 2. Materials and Methods

### 2.1. Study Design, Reference Hospitals, and Laboratories

This retrospective cross-sectional study was conducted between December 2023 and March 2025 at the King Salman Specialist Hospital, Ha’il, Saudi Arabia. The King Salman Specialist Hospital (KSSH) is certified and accredited by the Saudi Central Board for Accreditation of Healthcare Institutions (CBAHI) (Ref. No. HAL/MOH/HO5/34213) and, along with the Ha’il Health Regional Laboratory (HHRL) which is also certified and accredited by the CBAHI (Code 2739), constitutes a major cluster for healthcare diagnostic centers that receive samples for testing.

### 2.2. Patients’ Demographics and IRB Approval

We have enrolled records of 805 patients (329 males and 476 females) who presented with symptoms suggestive of upper gastrointestinal (GI) disorders. The age range extended from 20 to 80 years, and participants were recruited consecutively from the endoscopy unit or those who had stayed for a day or less to avoid hospital-associated infections. Each patient provided written informed consent (blank attached), and the study protocol was approved by the Institutional Review Board (Approval No. Log 2024-120, December 2024, University of Ha’il REC -H-2024-941, dated REC 4112024). Ethical procedures followed the guidelines set forth in the Declaration of Helsinki, ensuring the protection of participants’ rights and confidentiality.

### 2.3. Histopathologic Examinations

Upon enrollment, patients underwent a standardized diagnostic evaluation, including demographic data collection and a physical examination. Endoscopic investigations were performed by experienced gastroenterologists, who obtained gastric biopsy samples from the antrum and body regions to assess *H. pylori* infection and to evaluate histopathological changes. All biopsy specimens were promptly fixed in a 37–40% commercial formaldehyde solution (formalin), processed using conventional histological protocols, and embedded in paraffin. Tissue blocks were sectioned at 3–5 µm thickness on a rotary microtome, mounted on glass slides, deparaffinized with an organic solvent, and rehydrated in a graded series of alcohol baths.

For routine histopathological examinations, Harris’ hematoxylin and eosin (H&E) was employed. The H&E staining procedure involved applying hematoxylin (combined with a metallic mordant), differentiation in a weak acid solution, and subsequent bluing in an alkaline medium, followed by counterstaining with eosin. To detect *H. pylori*, Giemsa staining was utilized, preparing a working solution by mixing 40 mL of Giemsa stock (4 g Giemsa powder dissolved in 250 mL glycerol and 250 mL methanol) with 60 mL of distilled water. Stained slides were independently reviewed by an experienced gastrointestinal pathologist, who confirmed *H. pylori* only when distinctive comma- or S-shaped bacilli (approximately 2–4 μm in length and 0.5–1 μm in thickness) were identified on the mucosal surface or round pleomorphic cells within the mucus layer, typically forming small colonies. Cases with isolated or morphologically ambiguous organisms were classified as negative. Histological inflammation was graded on the basis of the number and distribution of polymorphonuclear neutrophils (PMNs) in the lamina propria. Moderate or severe inflammatory activity was defined by widespread PMN infiltration—often invading the glandular epithelium or forming microabscesses—whereas mild activity indicated a focal or limited presence of PMNs.

### 2.4. Statistical Analysis

Statistical analyses were carried out using IBM SPSS (version 23, IBM Corp., Armonk, NY). Continuous variables were summarized as means (± standard deviation) or medians (with interquartile ranges), while categorical data were presented as frequencies and percentages. The associations among *H. pylori* infection, demographic factors, and histopathological findings were examined using Chi-square or Fisher’s exact tests, with odds ratios (OR) and 95% confidence intervals (CI) calculated where appropriate. A two-tailed *p*-value < 0.05 was deemed statistically significant. This approach enabled a comprehensive evaluation of *H. pylori*’s prevalence, its relationship to patient characteristics, and its impact on gastric histopathology.

## 3. Results

### 3.1. Prevalence of H. pylori Infection

A total of 805 patients were included in this study, comprising 329 males (40.9%) and 476 females (59.1%). The prevalence of *Helicobacter pylori* infection was significantly high across all age groups. Among males aged 20–39 years, 209 (87.1%) tested positive for *H. pylori*, increasing to 81 (94.2%) in the 40–59 age group and reaching 3 (100.0%) in the 60–80 age group. Similarly, among females aged 20–39 years, 329 (91.9%) were infected, while in the 40–59 age group, 105 (89.0%) tested positive. No cases of *H. pylori* infection were recorded in females aged 60–80 years (Table 1).

### 3.2. Chronic Gastrointestinal Conditions

Regarding overall chronic gastrointestinal conditions, for clarity and specificity of the disorder with respect to internal organs, we have divided them into overall, major, and specific diseases to specifically identify the factors and rates of each. In the primary screening, nearly all patients exhibited some form of complications with 99.9% (*n* = 726) diagnosed with at least one chronic gastrointestinal condition, leaving only one patient without any recorded complications, which is not taken into consideration because of the low sample size. Lymphoma was observed in a single case, while metaplasia was identified in six (0.8%) patients. Chronic gastritis, a common pathologic diagnosis associated with *H. pylori* infection, was detected in 140 (19.3%) cases (Table 1).

Other pathological findings included gastric atrophy in two cases, gastric lipomas in six (0.8%) cases, and gastric polyps in five (0.7%) cases (Table 1). These findings indicate that while *H. pylori* infection is prevalent, progression to more severe conditions such as lymphoma and metaplasia remains relatively rare.

### 3.3. Symptoms Associated with H. pylori Infection

**Figure 1:**
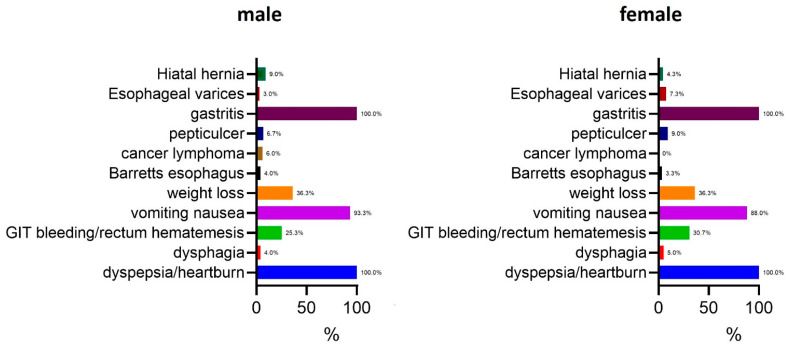
Prevalence of gastrointestinal symptoms in male and female patients diagnosed with *H. pylori* infection. Data are presented as percentages of affected individuals within each gender group.

The most prevalent pathologic findings diagnosed in both genders was gastritis, affecting 100.0% of the participants. Dyspepsia and heartburn were also universally reported in both males and females (100.0%). Other common symptoms included vomiting and nausea, which were present in 93.3% of males and 88.0% of females. Weight loss was equally prevalent in both genders, reported in 36.3% of cases. Gastrointestinal (GIT) bleeding or rectal hematemesis was more frequently observed in females (30.7%) compared with males (25.3%). Peptic ulcers were identified in 6.7% of males and 9.0% of females, while esophageal varices were slightly more common in females (7.3%) than in males (3.0%). Cancer lymphoma was observed in 6.0% of males and 9.0% of females, whereas Barrett’s esophagus was detected in 4.0% of males and 3.3% of females. Hiatal hernia was the least frequently reported symptom, occurring in 9.0% of males and 4.3% of females. Dysphagia was present in 4.0% of males and 5.0% of females (Figure 1).

Overall, these findings suggest that while gastritis and dyspepsia are universally present in *H. pylori*-infected individuals, gender-based differences exist in the prevalence of certain gastrointestinal complications.

### 3.4. Histopathological Findings

Histopathological analysis of gastric biopsies revealed varying degrees of inflammation and structural changes associated with *Helicobacter pylori* infection.

**Figure 2:**
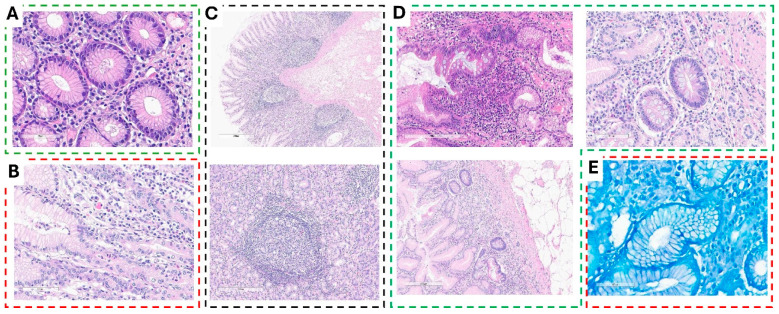
Histopathological features of *Helicobacter pylori*-associated gastric pathology. (Standard colors; Figures 50–100 µL) (**A**) Chronic inflammation with moderate to marked inflammatory infiltrate. H&E-stained gastric mucosa show preserved glandular architecture with an intact epithelium and mononuclear inflammatory cell infiltration in the lamina propria, indicating chronic inflammation. (**B**) Chronic active gastritis with potential *H. pylori* infection. Distorted glands and foveolar epithelium with dense lymphoplasmacytic infiltrate and scattered neutrophils, signifying active inflammation. Small curved or spiral-shaped organisms suggestive of *H. pylori* are present. (**C**) Chronic active gastritis with reactive lymphoid hyperplasia. The gastric biopsy reveals an intact foveolar epithelium, mild glandular distortion, and dense inflammatory infiltrate. Prominent lymphoid aggregates with germinal centers confirm reactive lymphoid hyperplasia. (**D**) Chronic active gastritis with glandular distortion and surface erosion. Sections display glandular atrophy, epithelial erosion, and intestinal metaplasia with reactive nuclear changes. The lamina propria is heavily infiltrated with inflammatory cells, consistent with *H. pylori*-associated gastritis. (**E**) Chronic gastritis with abundant *H. pylori*. Giemsa stain highlights curved, spiral-shaped *H. pylori* organisms colonizing the surface epithelium and mucus layer.

Chronic inflammation with moderate to marked inflammatory infiltrate was evident in multiple biopsy specimens. H&E staining of the gastric mucosa demonstrated a normal glandular architecture lined with an intact epithelium. However, the lamina propria exhibited moderate to marked infiltration of mononuclear inflammatory cells, predominantly lymphocytes and plasma cells, indicative of chronic inflammation (Figure 2A). In cases of chronic active gastritis with potential *H. pylori* infection, biopsies showed glandular distortion along with foveolar epithelial irregularities. The lamina propria was densely infiltrated with a mixed inflammatory cell population, primarily lymphocytes and plasma cells, accompanied by scattered neutrophils, confirming active inflammation. Notably, small curved or spiral-shaped organisms were observed on the mucosal surface, suggesting the presence of *H. pylori* (Figure 2B).

Sections of chronic active gastritis with reactive lymphoid hyperplasia, frequently associated with *H. pylori* infection, demonstrated an intact foveolar epithelium with no significant erosion or ulceration. Mild architectural distortion of the gastric glands was observed, along with a dense inflammatory infiltrate of lymphocytes and plasma cells in the lamina propria. Additionally, neutrophilic infiltration signified active inflammation. Prominent lymphoid aggregates with well-formed germinal centers were present within the lamina propria and submucosa, confirming reactive lymphoid hyperplasia (Figure 2C). In more severe cases, chronic active gastritis with glandular distortion and surface erosion, suggestive of *H. pylori*-associated gastritis, was identified. These biopsies exhibited significant glandular architectural distortion, along with signs of glandular atrophy. The surface epithelium displayed erosion and damage, accompanied by features of intestinal metaplasia and reactive epithelial alterations such as nuclear enlargement and hyperchromasia. A dense inflammatory infiltrate populated the lamina propria, reinforcing the diagnosis of chronic active gastritis with surface damage (Figure 2D). Confirming the presence of *H. pylori*, Giemsa-stained gastric biopsies revealed numerous pleomorphic, curved, spiral-shaped microorganisms adhering to the surface epithelium and within the mucosal layer, further supporting the diagnosis of *H. pylori*-associated chronic gastritis with abundant bacterial colonization (Figure 2E).

These findings highlight the spectrum of histopathological alterations observed in *H. pylori*-infected gastric mucosa, ranging from chronic inflammation to architectural distortion and bacterial colonization.

### 3.5. Statistical Analysis of Gastrointestinal Complications

Gastrointestinal complications were highly prevalent among *H. pylori*-infected individuals, with 770 (95.7%) of cases reporting at least one complication. No significant correlation was observed between gender and the likelihood of developing gastrointestinal complications (OR = 0.963; 95% CI: 0.482–1.922) (Supplementary Table S1).

Lymphoid aggregates, a key histopathological feature of *H. pylori*-induced gastritis, were present in 749 (93.0%) of cases, but their occurrence did not significantly differ between males and females (OR = 1.276; 95% CI: 0.740–2.200) (Supplementary Table S2). Similarly, lymphoma was detected in only one patient, making statistical comparisons unreliable (Supplementary Table S3).

Metaplasia, a potential precursor to gastric malignancy, was observed in six (0.8%) cases, with no significant gender-based risk difference (OR = 0.343; 95% CI: 0.062–1.883) (Supplementary Table S4). Chronic gastritis was diagnosed in 140 (19.3%) of the participants, but no significant gender correlation was found (OR = 0.888; 95% CI: 0.615–1.282) (Table 2).

Acute gastritis was diagnosed in 19 (2.4%) cases, again without any notable gender difference (OR = 0.949; 95% CI: 0.378–2.386) (Supplementary Table S5). Atrophy, a histological marker of gastric mucosal damage, was found in only 2 (0.3%) of participants, with no statistically significant correlation with gender (OR = 0.691; 95% CI: 0.043–11.079) (Supplementary Table S6).

The overall prevalence of *H. pylori* infection in the study population was 727 (90.3%), with a slightly higher infection rate in females (91.2%) compared with males (89.0%). However, this difference was not statistically significant (OR = 1.270; 95% CI: 0.794–2.030) (Table 3).

Gastric lipomas were identified in six (0.8%) cases, with no significant gender association (OR = 1.386; 95% CI: 0.252–7.609) (Supplementary Table S7). Similarly, gastric polyps were detected in five (0.7%) patients, with no significant gender correlation (OR = 2.780; 95% CI: 0.309–24.982) (Supplementary Table S8).

Overall, while *H. pylori* infection was widespread and associated with a high prevalence of chronic gastritis, most advanced gastrointestinal pathologies, including metaplasia, atrophy, lymphoma, and gastric tumors, remained rare, with no significant gender-based differences.

## 4. Discussion

This study provides meaningful perspectives on the multifaceted epidemiology, clinical features, and histopathologics of *H. pylori*. By interpreting our findings through the lens of established literature, we can better appreciate how these results inform both clinical care and broader public health efforts. Our observations, which align with numerous PubMed-indexed investigations, underscore *H. pylori*’s global importance and the considerable burden it imposes in terms of disease severity and mortality.

One of the most prominent observations is the considerable prevalence of *H. pylori* across various age categories in our cohort. Among men aged 20–39 years, 87.1% tested positive, rising to 94.2% in the 40–59 bracket, and all of the few patients aged 60–80—although the older group had a very few sample sizes in both genders and was not statistically significant. Similarly, women aged 20–59 showed infection rates between 89.0% and 91.9%, whereas none tested positive beyond 60 years in our study population. This level and increase with age are typical of *H.pylori*’s prevalence in developing countries [17]. Saudi Arabia has become an economic hub attracting jobseekers from diverse countries and is becoming one of the most desired destinations globally and in the region. It is consistent with known factors for contracting the organism, including consumption of restaurant food, meat, nonfiltered water, and smoking habits, mostly among youth in the region. However, its positivity in youth through to old age is a trend largely consistent with past epidemiological reports indicating that *H. pylori* is often acquired during childhood and can persist, remaining dormant intracellularly into later stages of life [18]. Nevertheless, the lack of cases in older women may point to localized demographic patterns, health-seeking habits in this subgroup, or the most reasonable possibility of smaller sample sizes in that age range.

On the clinical front, we noted that nearly every individual in our study experienced gastritis, dyspepsia, and heartburn. This widespread occurrence highlights the central role of *H. pylori* in upper gastrointestinal disorders [19]. Chronic inflammation triggered by *H. pylori* may also explain the frequency of coexisting symptoms such as vomiting, nausea, and, in some cases, gastrointestinal bleeding or hematemesis. Previous investigations have demonstrated how enduring inflammation can encourage disease progression if left unchecked [20]. We also detected autoimmune-like complications, aligning with existing literature that points to immunomodulatory processes driven by *H. pylori* in certain extragastric diseases [21]. Thus, despite advanced gastric lesions remaining rare in the region, the potential for malignant transformation in different conditions makes it imperative for continued vigorous eradication, therapy, and vigilant follow-up to avert severe disease outcomes.

Histologically, 19.3% of our patients showed evidence of chronic gastritis, while 2.4% had acute gastritis, reinforcing *H. pylori* as a primary source of sustained gastric mucosal inflammation. Nearly all examined cases (93.0%) displayed lymphoid aggregates, which are recognized as a hallmark of *H. pylori*-associated gastritis. Although MALT lymphoma is rare, we found one individual (0.1%) with this condition, illustrating the possibility that malignant transformations can arise in a small fraction of cases.

Interestingly, our results indicated that advanced pathologies, such as metaplasia (0.8%), atrophy (0.3%), and malignancy, were not widespread. Even so, identifying these changes in any subset of patients underscores the organism’s carcinogenic capability, consistent with *H. pylori*’s designation by the International Agency for Research on Cancer as a Class I carcinogen. It is essential to emphasize that chronic infection can move through Correa’s sequence, transitioning from chronic gastritis to atrophic gastritis, intestinal metaplasia, dysplasia, and eventually gastric adenocarcinoma [22]. Early detection and prompt treatment thus remain pivotal to curbing the risk of more advanced disease.

Overall, 90.3% of our study population tested positive for *H. pylori*, a figure comparable with findings from other developing regions where prevalence may approach or exceed 80%. However, the organism’s prevalence was linked more to the impact of diet [23], where mostly food, dairy, and veterinary products are prone to transmitting it [24]. Nonetheless, differences between age brackets in our data might stem from regional economic conditions, healthcare availability, hygiene standards, socioeconomic status, and cultural norms involving antibiotic practices, particularly in the widely practiced traditional dairy and veterinary farm products. Earlier research has indicated that crowding and poor sanitation in low-income settings can facilitate both family and community-level spread [25]. Meanwhile, countries with improved public health infrastructure have documented decreasing *H. pylori* infection rates over the past few decades, reflecting the positive impact of better living conditions and broader antibiotic use. Nonetheless, the high rates of food and labor imports in markets are likely to have significant impact in foodborne zoonotic pathogens’ transmission dynamics.

Our finding of considerable chronic gastritis cases underscores the pressing need for effective *H. pylori* eradication strategies. As documented in previous research, embracing a test-and-treat model can substantially lower the incidence of peptic ulcers and related complications [26]. Achieving this outcome, however, requires patient compliance, clinicians following best-practice guidelines, and continual assessment of local resistance dynamics. In places where antibiotic resistance is escalating, quadruple regimens with bismuth or second-line agents like tetracycline, levofloxacin, or rifabutin may be justified if susceptibility data support their use. Regardless of the specific medication combination, proton pump inhibitors are an essential component because they elevate gastric pH, thereby boosting the effectiveness of antimicrobial agents.

We noted no statistically significant sex-based differences regarding major *H. pylori* complications such as metaplasia, atrophy, or lymphoma, although women had a slightly higher infection rate (91.2% vs. 89.0%) and a modestly greater incidence of gastrointestinal bleeding and esophageal varices. These findings contrast with some epidemiological datasets suggesting that men might be more prone to duodenal ulcers and gastric cancer [27,28]. Geographic elements, population attributes, or genetic factors may account for these inconsistencies. Our results indicate that, irrespective of gender, early identification and timely intervention are crucial to managing *H. pylori*-related disease effectively.

Histopathologic evaluation of infected gastric mucosa revealed an active inflammatory profile with infiltration by neutrophils and mononuclear cells. Lymphoid aggregates, indicative of *H. pylori*’s immunostimulatory activity, were abundant. MALT lymphoma was present in only one patient—while uncommon, it is recognized to regress after *H. pylori*’s eradication. Although we encountered minimal advanced precancerous transformations, any trace of atrophy or metaplasia in these samples underscores the need to track patients who may be at heightened risk over time. According to Correa’s model, further histopathological changes, such as incomplete intestinal metaplasia, significantly increase the likelihood of developing gastric adenocarcinoma [29].

A number of investigations spanning Asia, Africa, and Latin America have demonstrated *H. pylori*’s pivotal influence on chronic gastric inflammation and the risk of peptic ulcers [30,31]. Notably, stronger public health infrastructure in some countries has been linked with falling *H. pylori* prevalence. Our findings—featuring widespread infection rates across key demographic segments—mirror similar data from the Middle East and North Africa, where hygiene factors, local customs, and transmission within households are crucial in shaping infection patterns.

Also noteworthy is that the comparatively slightly lower incidence of advanced lesions in our sample parallels observations from other areas of the developing world. High *H. pylori* prevalence does not always equate to a proportional escalation in malignancies. Variables such as genetic predisposition [32], bacterial virulence genes (for instance, cagA and vacA), and local dietary habits [25,33] modulate the trajectory from infection to severe disease. Comparative studies in Southeast Asia and Eastern Europe highlight differences in clinical outcomes, even among populations with similar infection levels, indicating the multifactorial nature of the disease progression of *H. pylori* [34]. Furthermore, role of *H. pylori* in many chronic disorders is not yet well studied; for instance, it has been found as an independent risk for thyroid nodules in adults. These findings highlight the critically important aspect of this organism’s eradication, not only for internal disorders but also for its role in other debilitating diseases such as Grave’s and Hashimoto’s thyroiditis [35]. Because of the low iodine in drinking water and the well-known genetic predispositions in the region, it is plausible to suggest *H. pylori* as a risk for multi-syndromic sequalae of disorders.

## 5. Conclusions

This study reaffirms *H. pylori*’s widespread prevalence in our region and reveals the spectrum of clinical and histopathological outcomes. Although most patients did not progress to advanced conditions, the presence of even a small fraction of malignant or near-malignant cases emphasizes the importance of monitoring. These findings resonate with global data while offering specific insights into local risk profiles, including gender distribution and symptom patterns. They also underscore how lymphoid aggregates might point to possible lymphoproliferative processes. When integrated with the wealth of PubMed-indexed studies, these insights are poised to refine clinical guidelines, optimize patient care, and move us closer to significantly reducing the worldwide impact of *H. pylori*. The major limitation of the study was the analysis of single-center patients. The future inclusion of multiple centers would gain more insights into the disease mechanisms and transmission dynamics of the organism. In addition, enrollment of both inpatients and outpatients in national centers as well as community screening centers would reveal much more information.

## Figures and Tables

**Table 1:** Prevalence of *H. pylori* infection and chronic gastrointestinal diseases stratified by gender and age groups.

Variable	Gender	Age (years)	N (%)
*H. pylori* bacteria	Male (*n* = 329)	20–39	No: 31 (12.9%)
			Yes: 209 (87.1%)
		40–59	No: 5 (5.8%)
			Yes: 81 (94.2%)
		60–80	No: 0 (0.0%)
			Yes: 3 (100.0%)
	Female (*n* = 476)	20–39	No: 29 (8.1%)
			Yes: 329 (91.9%)
		40–59	No: 13 (11.0%)
			Yes: 105 (89.0%)
		60–80	No: 0 (0.0%)
			Yes: 0 (0.0%)
Overall chronic diseases	Major gastrointestinal complications		No: 1 (0.1%)
			Yes: 726 (99.9%)
	Lymphoma		No: 726 (99.9%)
			Yes: 1 (0.1%)
	Metaplasia		No: 721 (99.2%)
			Yes: 6 (0.8%)
	Chronic gastritis		No: 587 (80.7%)
			Yes: 140 (19.3%)
	Atrophy		No: 725 (99.7%)
			Yes: 2 (0.3%)
	Gastric lipoma		No: 721 (99.2%)
			Yes: 6 (0.8%)
	Gastric polyp		No: 722 (99.3%)
			Yes: 5 (0.7%)

**Table 2:** Distribution of chronic gastritis among *H. pylori*-infected individuals, stratified by gender and assessing the relationship between gender and chronic gastritis.

Gender	Chronic Gastritis (no)	Chronic Gastritis (yes)	Total	Risk Estimate	Value	95% Confidence Interval (Lower)	95% Confidence Interval (Upper)
Male	268	61	329	Odds ratio for gender (male/female)	0.888	0.615	1.282
Female	396	80	476	For cohort chronic gastritis = no	0.979	0.917	1.045
Total	664	141	805	For cohort chronic gastritis = yes	1.103	0.816	1.492
				No. of valid cases	805		

**Table 3:** Gender-based risk estimate of *Helicobacter pylori* infection.

Gender	*H. pylori* (No)	*H. pylori* (Yes)	Total	Risk Estimate	Value	95% Confidence Interval (Lower)	95% Confidence Interval (Upper)
Male	36	293	329	Odds ratio for gender (male/female)	1.270	0.794	2.030
Female	42	434	476	For cohort *H. pylori* = no	1.240	0.813	1.892
Total	78	727	805	For cohort *H. pylori* = yes	0.977	0.932	1.024
				No. of valid cases	805		

## Data Availability

Supplementary Tables S1–S8 indicated in the text (Results) are enclosed uploaded here with this manuscript.

## Supplementary Materials

The following supporting information can be downloaded at https://www.mdpi.com/article/10.3390/jcm14082643/s1.

## References

[1] Chen Y.C., Malfertheiner P., Yu H.T., Kuo C.L., Chang Y.Y., Meng F.T., Wu Y.-X., Hsiao J.-L., Chen M.-J., Lin K.-P. (2024). Global Prevalence of *Helicobacter pylori* Infection and Incidence of Gastric Cancer Between 1980 and 2022. Gastroenterology.

[2] Malfertheiner P., Camargo M.C., El-Omar E., Liou J.M., Peek R., Schulz C., Smith S.I., Suerbaum S. (2023). *Helicobacter pylori* infection. Nat. Rev. Dis. Primers..

[3] Carrasco G., Corvalan A.H. (2013). *Helicobacter pylori*-Induced Chronic Gastritis and Assessing Risks for Gastric Cancer. Gastroenterol. Res. Pract..

[4] Schöttker B., Adamu M.A., Weck M.N., Brenner H. (2012). *Helicobacter pylori* infection is strongly associated with gastric and duodenal ulcers in a large prospective study. Clin. Gastroenterol. Hepatol..

[5] Emmanuel B.N., Peter D.A., Peter M.O., Adedayo I.S., Olaifa K. (2024). *Helicobacter pylori* infection in Africa: Comprehensive insight into its pathogenesis, management, and future perspectives. J. UmmAl-Qura Univ. Appl. Sci..

[6] Ibrahim M.E. (2024). Epidemiology, pathogenicity, risk factors, and management of *Helicobacter pylori* infection in Saudi Arabia. Biomol. Biomed..

[7] Alsulaimany F.A.S., Awan Z.A., Almohamady A.M., Koumu M.I., Yaghmoor B.E., Elhady S.S., Elfaky M.A. (2020). Prevalence of *Helicobacter pylori* Infection and Diagnostic Methods in the Middle East and North Africa Region. Medicina.

[8] Gravina A.G., Priadko K., Ciamarra P., Granata L., Facchiano A., Miranda A., Dallio M., Federico A., Romano M. (2020). Extra-Gastric Manifestations of *Helicobacter pylori* Infection. J. Clin. Med..

[9] Pellicano R., Ianiro G., Fagoonee S., Settanni C.R., Gasbarrini A. (2020). Review: Extragastric diseases and *Helicobacter pylori*. Helicobacter.

[10] Iarc L. (1994). Schistosomes, liver flukes and *Helicobacter pylori*. IARC Monographs on the Evalutaion of Carcinogenic Risks to Humans.

[11] Feldman M., Friedman L.S., Brandt L.J. (2020). Sleisenger and Fordtran’s Gastrointestinal and Liver Disease E-Book: Pathophysiology, Diagnosis, Management.

[12] Wang L., Cao Z.M., Zhang L.L., Dai X.C., Liu Z.J., Zeng Y.X., Li X.Y., Wu Q.J., Lv W.L. (2022). *Helicobacter Pylori* and Autoimmune Diseases: Involving Multiple Systems. Front. Immunol..

[13] Hasni S.A. (2012). Role of *Helicobacter pylori* infection in autoimmune diseases. Curr. Opin. Rheumatol..

[14] Wang W.L., Xu X.J. (2019). Correlation Between *Helicobacter pylori* Infection and Crohn’s Disease: A Meta-Analysis. Eur. Rev. Med. Pharmacol. Sci..

[15] Costa L.C.M.C., das Graças Carvalho M., La Guárdia Custódio Pereira A.C., Teixeira Neto R.G., Andrade Figueiredo L.C., Barros-Pinheiro M. (2024). Diagnostic Methods for *Helicobacter pylori*. Med. Princ. Pract..

[16] Nakashima H., Kawahira H., Kawachi H., Sakaki N. (2018). Artificial intelligence diagnosis of *Helicobacter pylori* infection using blue laser imaging-bright and linked color imaging: A single-center prospective study. Ann. Gastroenterol..

[17] Woodward M., Morrison C., McColl K. (2000). An investigation into factors associated with *Helicobacter pylori* infection. J. Clin. Epidemiol..

[18] Yang Y.-J., Yang H.-B., Wu J.-J., Sheu B.-S. (2008). Persistent *H. pylori* colonization in early acquisition age of mice related with higher gastric sialylated Lewis x, IL-10, but lower interferon-γ expressions. J. Biomed. Sci..

[19] Moayyedi P., Forman D., Braunholtz D., Feltbower R., Crocombe W., Liptrott M., Axon A., Leeds HELP Study Group (2000). The proportion of upper gastrointestinal symptoms in the community associated with *Helicobacter pylori*, lifestyle factors, and nonsteroidal anti-inflammatory drugs. Am. J. Gastroenterol..

[20] Loosen S.H., Mertens A., Klein I., Leyh C., Krieg S., Kandler J., Luedde T., Roderburg C., Kostev K. (2024). Association between *Helicobacter pylori* and its eradication and the development of cancer. BMJ Open Gastroenterol..

[21] Hou Y., Sun W., Zhang C., Wang T., Guo X., Wu L., Qin L., Liu T. (2017). Meta-analysis of the correlation between *Helicobacter pylori* infection and autoimmune thyroid diseases. Oncotarget.

[22] Toh J.W.T., Wilson R.B. (2020). Pathways of Gastric Carcinogenesis, *Helicobacter pylori* Virulence and Interactions with Antioxidant Systems, Vitamin C and Phytochemicals. Int. J. Mol. Sci..

[23] He S., He X., Duan Y., Luo Y., Li Y., Li J., Li Y., Yang P., Wang Y., Xie J. (2024). The impact of diet, exercise, and sleep on *Helicobacter pylori* infection with different occupations: A cross-sectional study. BMC Infect. Dis..

[24] Elhariri M., Hamza D., Elhelw R., Hamza E. (2018). Occurrence of *cagA*^+^
*vacA s1a m1 i1 Helicobacter pylori* in farm animals in Egypt and ability to survive in experimentally contaminated UHT milk. Sci. Rep..

[25] Amaral O., Fernandes I., Veiga N., Pereira C., Chaves C., Nelas P., Silva D. (2017). Living Conditions and *Helicobacter pylori* in Adults. Biomed. Res. Int..

[26] Gisbert J.P., Calvet X. (2013). Helicobacter Pylori “Test-and-Treat” Strategy for Management of Dyspepsia: A Comprehensive Review. Clin. Transl. Gastroenterol..

[27] Luan X., Niu P., Wang W., Zhao L., Zhang X., Zhao D., Chen Y. (2022). Sex Disparity in Patients with Gastric Cancer: A Systematic Review and Meta-Analysis. J. Oncol..

[28] Arakawa H., Komatsu S., Kamiya H., Nishibeppu K., Ohashi T., Konishi H., Shiozaki A., Kubota T., Fujiwara H., Otsuji E. (2023). Differences of clinical features and outcomes between male and female elderly patients in gastric cancer. Sci. Rep..

[29] Jencks D.S., Adam J.D., Borum M.L., Koh J.M., Stephen S., Doman D.B. (2018). Overview of Current Concepts in Gastric Intestinal Metaplasia and Gastric Cancer. Gastroenterol. Hepatol..

[30] Graham D.Y., Lu H., Yamaoka Y. (2009). African, Asian or Indian enigma, the East Asian *Helicobacter pylori*: Facts or medical myths. J. Dig. Dis..

[31] Bustos-Fraga S., Salinas-Pinta M., Vicuña-Almeida Y., de Oliveira R.B., Baldeón-Rojas L. (2023). Prevalence of *Helicobacter pylori* genotypes: *cagA*, *vacA (m1)*, *vacA (s1)*, *babA2*, *dupA*, *iceA1*, *oipA* and their association with gastrointestinal diseases. A cross-sectional study in Quito-Ecuador. BMC Gastroenterol..

[32] Datta De D., Roychoudhury S. (2015). To be or not to be: The host genetic factor and beyond in *Helicobacter pylori* mediated gastro-duodenal diseases. World J. Gastroenterol..

[33] Roesler B.M., Rabelo-Gonçalves E.M., Zeitune J.M. (2014). Virulence Factors of *Helicobacter pylori*: A Review. Clin. Med. Insights Gastroenterol..

[34] Cavadas B., Leite M., Pedro N., Magalhães A.C., Melo J., Correia M., Máximo V., Camacho R., Fonseca N.A., Figueiredo C. (2021). Shedding Light on the African Enigma: In Vitro Testing of Homo sapiens-*Helicobacter pylori* Coevolution. Microorganisms.

[35] Di J., Ge Z., Xie Q., Kong D., Liu S., Wang P., Li J., Ning N., Qu W., Guo R. (2023). *Helicobacter pylori* infection increases the risk of thyroid nodules in adults of Northwest China. Front. Cell. Infect. Microbiol..

