# Supplementary material for: Clinical Pathologic Profiles of Helicobacter pylori Reveal Age-Specific Peaking with Concomitant Chronic Gastric Inflammation, Robust Immunity, and Tissue Alterations Implying Potential Predisposition to Malignancy in Ha’il, Saudi Arabia
Source: J Clin Med. 2025 Apr 11;14(8):2643. doi: 10.3390/jcm14082643 (PMC12028268; doi:10.3390/jcm14082643)
Supplement: Supplementary file 1 [file jcm-14-02643-s001.zip › jcm-3563327-supplementary.pdf]

Supplementary Table S1. Distribution of gastrointestinal complications among H. pylori-infected individuals, stratified by gender and assessing the relationship between gender and gastrointestinal complications.

Gender	Gastrointestinal complications (No)	Gastrointestinal complications (Yes)	Total	Risk Estimate	Value	95% Confidence Interval (Lower)	95% Confidence Interval (Upper)
**Male**	14	315	329	**Odds Ratio for Gender (Male / Female)**	0.963	0.482	1.922
**Female**	21	455	476	**For Cohort Gastrointestinal complications = No**	0.965	0.498	1.869
**Total**	35	770	805	**For Cohort Gastrointestinal complications = Yes**	1.002	0.972	1.032
				**N of Valid Cases**	**805**		

Supplementary Table S2. Distribution of Lymphoid aggregate among H. pylori-infected individuals, stratified by gender and assessing the relationship between gender and Lymphoid aggregate.

Gender	Lymphoid aggregate (No)	Lymphoid aggregate (Yes)	Total	Risk Estimate	Value	95% Confidence Interval (Lower)	95% Confidence Interval (Upper)
**Male**	26	303	329	**Odds Ratio for Gender (Male / Female)**	1.276	0.740	2.200
**Female**	30	446	476	**For Cohort Lymphoid aggregate = No**	1.254	0.756	2.080
**Total**	56	749	805	**For Cohort Lymphoid aggregate = Yes**	0.983	0.945	1.022
				**N of Valid Cases**	805		

Supplementary Table S3. Distribution of Lymphoma among H. pylori-infected individuals, stratified by gender and assessing the relationship between gender and Lymphoma.

Gender	Lymphoma (No)	Lymphoma (Yes)	Total	Risk Estimate	Value	95% Confidence Interval (Lower)	95% Confidence Interval (Upper)
**Male**	328	1	329	**Odds Ratio for Gender (Male / Female)**	-	-	-
**Female**	476	0	476	**For Cohort Lymphoma = No**	0.997	0.991	1.003
**Total**	804	1	805	**For Cohort Lymphoma = Yes**	-	-	-
				**N of Valid Cases**	805		

Supplementary Table S4. Distribution of Metaplasia among H. pylori-infected individuals, stratified by gender and assessing the relationship between gender and Metaplasia.

Gender	Metaplasia (No)	Metaplasia (Yes)	Total	Risk Estimate	Value	95% Confidence Interval (Lower)	95% Confidence Interval (Upper)
**Male**	325	4	329	**Odds Ratio for Gender (Male / Female)**	0.343	0.062	1.883
**Female**	474	2	476	**For Cohort Metaplasia = No**	0.992	0.979	1.005
**Total**	799	6	805	**For Cohort Metaplasia = Yes**	2.894	0.533	15.706
				**N of Valid Cases**	805		

Supplementary Table S5. Distribution of Acute gastritis among H. pylori-infected individuals, stratified by gender and assessing the relationship between gender and Acute gastritis.

Gender	Acute gastritis (No)	Acute gastritis (Yes)	Total	Risk Estimate	Value	95% Confidence Interval (Lower)	95% Confidence Interval (Upper)
**Male**	321	8	329	**Odds Ratio for Gender (Male / Female)**	0.949	0.378	2.386
**Female**	465	11	476	**For Cohort Acute gastritis = No**	0.999	0.977	1.021
**Total**	786	19	805	**For Cohort Acute gastritis = Yes**	1.052	0.428	2.588
				**N of Valid Cases**	805		

Supplementary Table S6. Distribution of Atrophy among H. pylori-infected individuals, stratified by gender and assessing the relationship between gender and Atrophy.

Gender	Atrophy (No)	Atrophy (Yes)	Total	Risk Estimate	Value	95% Confidence Interval (Lower)	95% Confidence Interval (Upper)
**Male**	328	1	329	**Odds Ratio for Gender (Male / Female)**	0.691	0.043	11.079
**Female**	475	1	476	**For Cohort Atrophy = No**	0.999	0.992	1.006
**Total**	803	2	805	**For CohortAtrophy = Yes**	1.447	0.091	23.049
				**N of Valid Cases**	805		

Supplementary Table S7. Distribution of Gastric lipomas among H. pylori-infected individuals, stratified by gender and assessing the relationship between gender and Gastric lipomas.

Gender	Gastric lipomas (No)	Gastric lipomas (Yes)	Total	Risk Estimate	Value	95% Confidence Interval (Lower)	95% Confidence Interval (Upper)
**Male**	327	2	329	**Odds Ratio for Gender (Male / Female)**	1.386	0.252	7.609
**Female**	472	4	476	**For Cohort Gastric lipomas = No**	1.002	0.991	1.014
**Total**	799	6	805	**For Cohort Gastric lipomas = Yes**	0.723	0.133	3.927
				**N of Valid Cases**	805		

Supplementary Table S8. Distribution of Gastric polyps among H. pylori-infected individuals, stratified by gender and assessing the relationship between gender and Gastric polyps.

Gender	Gastric polyps (No)	Gastric polyps (Yes)	Total	Risk Estimate	Value	95% Confidence Interval (Lower)	95% Confidence Interval (Upper)
**Male**	328	1	329	**Odds Ratio for Gender (Male / Female)**	2.780	0.309	24.982
**Female**	472	4	476	**For Cohort Gastric polyps = No**	1.005	0.995	1.016
**Total**	800	5	805	**For Cohort Gastric polyps = Yes**	0.362	0.041	3.222
				**N of Valid Cases**	805		
